# Snail heterogeneity in clear cell renal cell carcinoma

**DOI:** 10.1186/s12885-016-2237-x

**Published:** 2016-03-08

**Authors:** Laura Zaldumbide, Asier Erramuzpe, Rosa Guarch, Rafael Pulido, Jesús M. Cortés, José I. López

**Affiliations:** Department of Pathology, Cruces University Hospital, University of the Basque Country (UPV/EHU), Plaza de Cruces s/n, 48903 Barakaldo, Bizkaia Spain; Quantitative Biomedicine Unit, Biocruces Health Research Institute, Barakaldo, Bizkaia Spain; Department of Pathology, Complejo Hospitalario de Navarra-Hospital Virgen del Camino, Pamplona, Navarra Spain; Biomarkers in Cancer Unit, Biocruces Health Research Institute, Barakaldo, Bizkaia Spain; Ikerbasque, Basque Foundation for Science, Bilbao, Bizkaia Spain; Department of Cell Biology and Histology, University of the Basque Country (UPV/EHU), 48940 Leioa, Spain

**Keywords:** Clear cell renal cell carcinoma, Intratumor heterogeneity, Epithelial to mesenchymal transition, Immunohistochemistry, Snail, Tumor sampling

## Abstract

**Background:**

Intratumor heterogeneity may be responsible of the unpredictable aggressive clinical behavior that some clear cell renal cell carcinomas display. This clinical uncertainty may be caused by insufficient sampling, leaving out of histological analysis foci of high grade tumor areas. Although molecular approaches are providing important information on renal intratumor heterogeneity, a focus on this topic from the practicing pathologist’ perspective is still pending.

**Methods:**

Four distant tumor areas of 40 organ-confined clear cell renal cell carcinomas were selected for histopathological and immunohistochemical evaluation. Tumor size, cell type (clear/granular), Fuhrman’s grade, Staging, as well as immunostaining with Snail, ZEB1, Twist, Vimentin, E-cadherin, β-catenin, PTEN, p-Akt, p110α, and SETD2, were analyzed for intratumor heterogeneity using a classification and regression tree algorithm.

**Results:**

Cell type and Fuhrman’s grade were heterogeneous in 12.5 and 60 % of the tumors, respectively. If cell type was homogeneous (clear cell) then the tumors were low-grade in 88.57 % of cases. Immunostaining heterogeneity was significant in the series and oscillated between 15 % for p110α and 80 % for Snail. When Snail immunostaining was homogeneous the tumor was histologically homogeneous in 100 % of cases. If Snail was heterogeneous, the tumor was heterogeneous in 75 % of the cases. Average tumor diameter was 4.3 cm. Tumors larger than 3.7 cm were heterogeneous for Vimentin immunostaining in 72.5 % of cases. Tumors displaying negative immunostaining for both ZEB1 and Twist were low grade in 100 % of the cases.

**Conclusions:**

Intratumor heterogeneity is a common event in clear cell renal cell carcinoma, which can be monitored by immunohistochemistry in routine practice. Snail seems to be particularly useful in the identification of intratumor heterogeneity. The suitability of current sampling protocols in renal cancer is discussed.

## Background

Renal cancer is among the most common malignancies in men and women in Western countries, with more than 61000 estimated new cases in the United States in 2015 [1]. Clear cell renal cell carcinoma (CCRCC) is the most frequent renal cancer histologic subtype, accounting for about 70 % of renal carcinomas [2]. CCRCC management is problematic for urologists and oncologists. This neoplasm follows a quite unpredictable clinical course and usually displays resistance to radio- and chemotherapy, surgery being the only effective treatment to date.

CCRCC is a paradigm of an intrinsically heterogeneous neoplasm and most problems with its clinical management rely on this point. Intratumor heterogeneity (ITH) in CCRCC follows a spatial and temporal branched pattern [3–5], with multiple cell clones evolving independently from each other during tumor evolution. This fact reflects the complexity of tumor biology [6] and adds further difficulties to define effective targeted treatments against this neoplasm [7]. CCRCC ITH has been specifically studied in the last years from a molecular perspective [3, 4, 7–9]. However, there are few studies addressing the problem from a clinical routine approach [10–12].

The aim of this study was to evaluate the ITH of the expression of several epithelial to mesenchymal transition (EMT) markers and PI3K/PTEN/Akt-pathway markers, as assessed by immunohistochemistry, and its relationship with standard histopathological prognosis parameters in a series of 40 CCRCC collected prospectively.

## Methods

The authors declare that all the experiments carried out in this study comply with current Spanish and European Union legal regulations, and have been approved by the Ethical and Scientific Committees of the Basque Country Public Health System (Osakidetza) (CEIC-E 2015/060).

Forty organ-confined CCRCCs from two Spanish medical institutions were included prospectively in the study from September 2011 to June 2012. Cases showing gross hemorrhage, necrosis or hard whitish areas indicative of sarcomatoid transformation were excluded. All the cases were collected prospectively, diagnosed and classified by the same pathologist in each hospital following similar criteria. Clinical data were retrieved from medical histories.

On gross examination, four distant zones were sampled in each tumor and then formalin-fixed and paraffin-embedded following routine methods. Whenever possible, samples were obtained in the cardinal points of the largest tumor slice and had to be similar to the naked eye. Once the resulting hematoxylin-eosin (HE) slides were histologically reviewed, a 2.5 mm-in-diameter tumor sample was selected in each block to build tissue microarrays (TMA). TMA were performed with the resulting 160 tumor samples (4 areas each case, 40 cases). Samples were placed randomly in the TMA to assure blind evaluation. Each TMA contained an internal control.

**Fig. 1:**
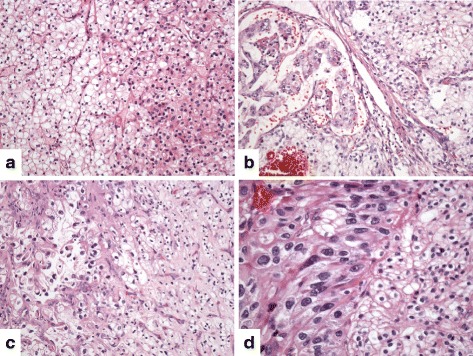
Intratumor heterogeneity in adjacent tumor areas of clear cell renal cell carcinomas on routine hematoxylin-eosin slides. **a** Clear and granular cells, **b** Papillary and solid cell tumor architecture, **c** High (papillary) and low (solid) Fuhrman’s grades, **d** High and low Fuhrman’s grades in clear cells (solid pattern)

Standard histopathologic data were evaluated on HE stained slides (Fig. 1), including cell-type (clear vs. granular), Fuhrman’s grade [classical (G1, G2, G3 and G4) and grouped in low (G1/2) and high (G3/4) grades], tumor diameter, and pT UICC staging (pT1a, pT1b and pT2). Tumors were considered histologically heterogeneous when at least one of the four samples of each tumor in the TMA had different cell type and/or grade.

**Fig. 2:**
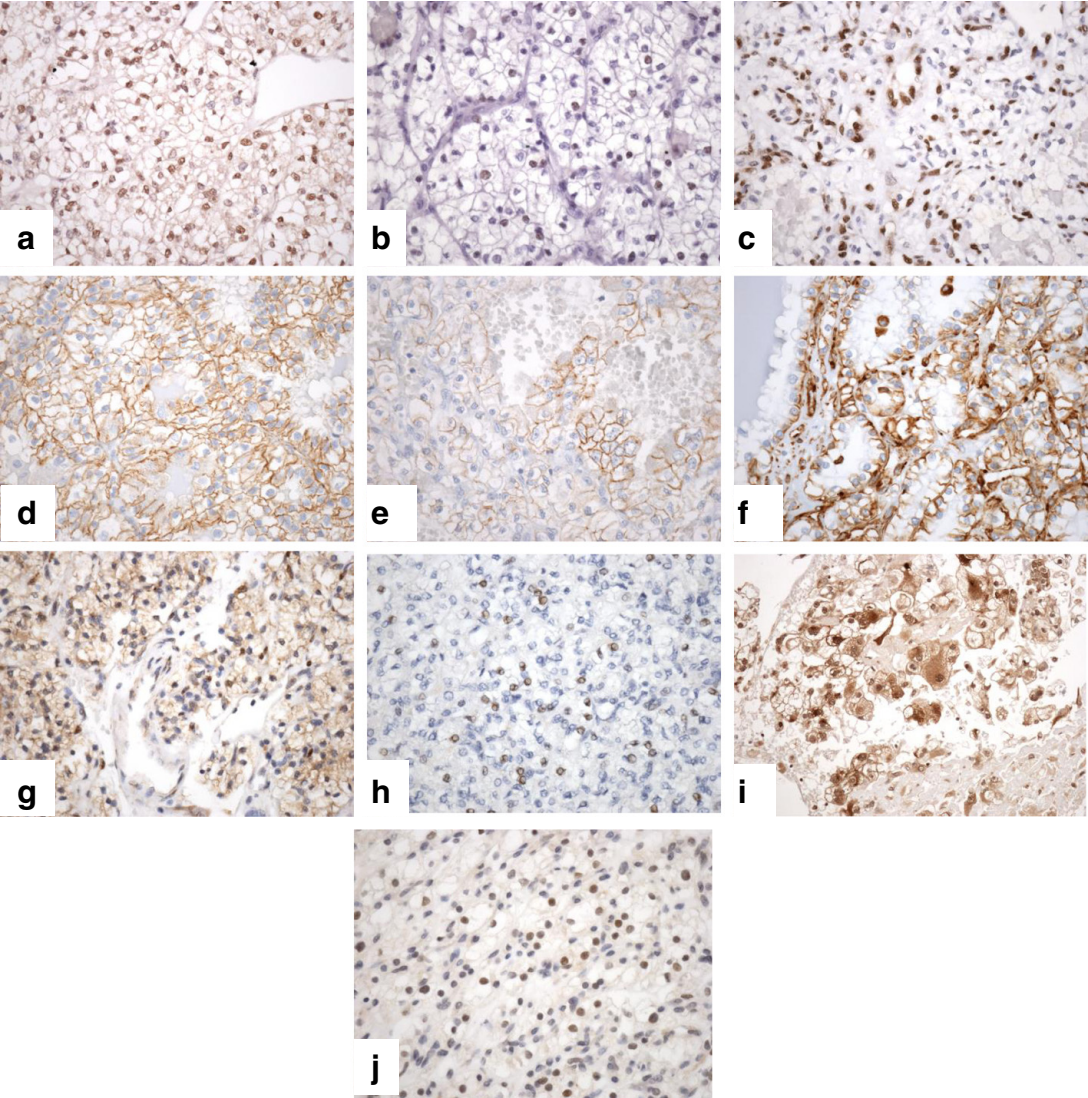
Immunohistochemical pattern in clear cell renal cell carcinomas displaying nuclear and/or cytoplasmic and/or membranous staining. **a** Snail, **b** Twist, **c** ZEB1, **d** β-catenin, **e** E-cadherin, **f** Vimentin, **g** PTEN, **h** p110α, **i** p-Akt, **j** SETD2

Immunohistochemical staining was performed in automated immunostainers (EnVision FLEX, Dako Autostainer Plus, Dako, Glostrup, Denmark; and BenchMark Ultra, Ventana Medical Systems, Tucson, AZ, USA) following routine methods. Tris-EDTA was used for antigen retrieval in all cases. Negative controls were slides not exposed to the primary antibody, and these were incubated in PBS and then processed under the same conditions as the test slides. The analysis was performed using a Nikon Eclipse 80i microscope (Tokyo, Japan). A panel of antibodies against EMT markers (Snail, Twist, ZEB1, β-catenin, E-cadherin, and Vimentin), PI3K/PTEN/Akt pathway-related markers (PTEN, p110α, p-Akt), as well as SETD2, was tested (Fig. 2). Table 1 shows the source and working dilutions of these antibodies. Heterogeneous immunostaining category was assigned when positive and negative immunostainings were detected among the four samples of each tumor. Cases were considered homogeneous when the four samples of each tumor displayed the same immunohistochemical profile, and could be positive or negative.

**Table 1:** List of antibodies tested in the study

Ab	Source/code			Dilution
Snail	Abcam, ab180714	polyclonal	↑ pH	1:75
Twist	Santa Cruz, sc15393	polyclonal	↓ pH	1:250
ZEB1	Santa Cruz, sc25388	polyclonal	↑ pH	1:100
β-catenin	Cell Marque, 760-4242	monoclonal	↑ pH	prediluted
E-cadherin	Roche, Ventana, 790-4497	monoclonal	↑ pH	prediluted
Vimentin	Roche, Ventana, 790-2917	monoclonal	↑ pH	prediluted
PTEN (6H2)	Dako, M3627	monoclonal	↑ pH	1:50
p110α	Pierce, PA5-27192	polyclonal	↓ pH	1:500
Akt	Life Technologies, 700392	monoclonal	↑ pH	1:500
SETD2	Pierce, PA5-34934	polyclonal	↑ pH	1:500

In a preliminary analysis, an independent 2-group *t* test was performed to check if age, sex or tumor size were different in the two conditions, heterogeneous vs. homogeneous (*t* test function in R, r-project.org). Afterwards, we performed classification and attribute selection methods for ITH. First, we chose ITH as the response variable to perform classification, coding it in a binary variable with two values, to be heterogeneous or not. Next, a classification and regression tree (CART) algorithm was performed using a 10-fold stratified cross-validation for testing. In particular, the CART algorithm was applied using the Waikato Environment for Knowledge Analysis (WEKA) [13]. Clinical, histological and immunohistochemical descriptors were selected to obtain the classification rules for the heterogeneity response; in particular, we used age, sex, simple Fuhrman’s grade (grades from 1 to 4), grouped Fuhrman’s grade [low grade (G1/2) and high grade (G3/4)], tumor diameter, pT staging, cell type (clear vs. granular) and immunostaining results of all the antibodies tested.

The use of WEKA also allowed us to discriminate the relevant descriptors (those really affecting the classification performance) from the irrelevant ones (those which did not contribute to the classification performance). Attribute selection was performed using three different algorithms (a best-first search, a rank-search, and a random-search) and the results did not differ between the three methods.

## Results

There was a male predominance in the series (30 M/10 F) with an average age of 62.3 years (range, 33–88). Tumor diameter oscillated between 1 and 10 cm (average, 4.3 cm). Following the aforementioned methodology, all the cases were organ confined and pT distribution was as follows: pT1a, 21 cases (52.5 %); pT1b, 17 cases (42.5 %); pT2: 2 cases (5 %). On HE stained sections 35 cases (87.5 %) were homogeneous and 5 heterogeneous (12.5 %) with respect to cell type. All the homogeneous cases were composed of conventional clear cells. Heterogeneous cases combined clear and granular cells. No case composed exclusively by granular cells was identified. Fuhrman’s grade [14] was homogeneous in the four samples in 16 cases (40 %), 11 of them being G1 and 5 G2, and heterogeneous in 24 cases (60 %). Grouped grading distribution displayed 32 homogeneous (80 %) and 8 heterogeneous (20 %) cases. All homogeneous cases in the series were low grade (G1/2) tumors (100 %).

Immunohistochemical results for ITH are summarized in Table 2. Briefly, Snail immunostaining was heterogeneous in 80 % of cases (32/40), Twist in 40 % (16/40), ZEB-1 in 62.5 % (25/40), β-catenin in 37.5 % (15/40), E-cadherin in 37.5 % (15/40), Vimentin in 50 % (20/40), PTEN in 35 % (14/40), p110α in 15 % (6/40), p-Akt in 30 % (12/40), and SETD2 in 42.5 % (17/40). Immunostaining pattern did not show any difference between G3 and G4 areas. Snail immunostaining showed a relation with Fuhrman’s grade: all high grade (G3/4) CCRCC were Snail heterogeneous and all Snail homogeneous CCRCC were low grade tumors (G1/2). Also, ZEB1 and Twist immunostainings were correlated with grade: if both ZEB1 and Twist immunostaining was negative, then tumor grade was low in 100 % of the cases.

**Table 2:** Immunohistochemical results

	Homogeneous		Heterogeneous
Abs	Positive	Negative	
Snail	8	0	32 (80 %)
Twist	1	23	16 (40 %)
ZEB-1	1	14	25 (62.5 %)
β-catenin	22	3	15 (37.5 %)
E-cadherin	0	25	15 (37.5 %)
Vimentin	18	2	20 (50 %)
PTEN (6H2)	0	26	14 (35 %)
p110α	34	0	6 (15 %)
Akt	0	28	12 (30 %)
SETD2	23	0	17 (42.5 %)

We obtained three different classification results from applying the CART algorithm to different response variables (Table 3). Although in general the output of a CART algorithm might be a decision tree with multiple branches, for the particular situation of our data sets, trees only had 2 branches, resulting in only one determinant variable for the three particular situations. First, considering all possible descriptors and ITH as the response variable, the decision tree of the CART classification showed that the only classifier variable was Snail immunostaining. Thus, if Snail immunostaining was homogeneous, the tumor was histologically homogeneous in all cases. By contrast, if Snail immunostaining was heterogeneous, the tumor was histologically heterogeneous in 75 % of cases. Using this classification scheme, 80 % of the cases in the series were correctly classified. Any other variable, alone or in combination, was not relevant for ITH.

**Table 3:** CART classification results over three different response variables (first column) revealed three different determinant variables (second column)

Response variable	Determinant variable	Individual Correctness	Individual Correctness	Total Correctness
		1	2	
Grade Heterogeneity	Snail immunostaining	Snail het - > Grade het	Snail hom - > Grade hom	80 %
		75 % (24/32)	100 % (8/8)	(32/40)
Grade	Cell type	Clear cell (hom) - > Grade low (G1/G2)	Granular cell (het) - > Grade high (G3/G4)	87.5 %
		88.57 % (31/35)	80 % (4/5)	(35/40)
Diameter	Vimentin	Diam > 3.7 - > Vim het	Diam < =3.7 - > Vim hom	72.5 %
		72.72 % (16/22)	72.22 % (13/18)	(29/40)

Individual Correctness (columns 3 and 4) shows the classification performance with respect to each group of the response variable and Total Correctness (column 5) shows the overall classification performance applied to the entire dataset

A second CART classification was performed considering Grade as the response variable. In this case, cell type (clear vs. granular) variable was the best one to classify the response. If cell type was ‘clear’ (group 1 in Table 3), then Grade was classified as G1-G2 with 88.57 % of correct classified instances. If cell type was a mixture of ‘clear and granular’ (group 2 in Table 3), then the Grade was G3-G4 with 80 % of correctness. Using this classification scheme, 87.5 % of the cases in the series were correctly classified. Any other variable, alone or in combination, was not relevant for heterogeneity in Grade classification.

Finally, a third CART classification analysis was performed choosing tumor diameter, Grade, grouped Grade and cell type, and combinations of them, as descriptors, and the rest of variables as response variables. This analysis showed that Vimentin was the only variable classified with correctness higher than 66 %. The rest of variables gave a poorer performance. This analysis unveiled that those samples with a tumor diameter larger than 3.7 cm were heterogeneous for Vimentin immunostaining with an accuracy of 72.5 %.

## Discussion

ITH is an inherent phenomenon in carcinogenesis that is being intensively analyzed using massive sequencing tools in many human neoplasms, kidney included [3–5, 7–9, 15]. However, pathological studies related with ITH under daily routine conditions are scarce. At this respect, Nassar et al. [16] documented ITH in breast cancer by immunohistochemistry and highlighted the negative clinical impact of potential false negative immunohistochemical results obtained after a study performed in small, probably non representative, core biopsies. In renal cancer this is an important issue since renal tumors are frequently big in size, and current protocols of tumor sampling recommend the selection for microscopic study of one block per centimeter of tumor plus an additional sample of every suspicious area on naked eye [17–19]. Following these guidelines, more than 90 % of the neoplastic tissue in many renal tumors may escape the pathologist’s routine analysis. This fact makes difficult to quantify the nature and extent of ITH, if present, in most CCRCC. Although pathologists recognize by naked eye whitish hard areas related to sarcomatoid transformation, as well as hemorrhagic and/or necrotic foci, there is a significant amount of ITH hidden in apparently homogeneous areas, as recently reported [10–12]. Furthermore, an incomplete tumor sampling may justify the unexpected aggressive behavior that some low-grade CCRCC show [12]. The present work has shown the relationship between cell type and Grade in CCRCC, since a mixture of conventional clear and granular cells determines high tumor Grade (G3/G4) in more than 83 % of cases.

Two recent studies on CCRCC have contributed to know more about ITH from a practical perspective. The first one intended to identify how much information is lost in routine practice after following the official sampling protocols. For such a purpose, a total tumor sampling, an unsuitable strategy in daily routine, was performed in 47 prospective CCRCC, and the histological analysis revealed a significant higher number of high grade tumors than with conventional sampling [10]. This finding raises serious doubts about the appropriateness of currently accepted CCRCC sampling protocols, suggesting that in some histopathological examinations data potentially crucial for the patient might be ignored. The second study used data mining tools and showed that only CCRCC tumors with less than 3.8 cm in diameter are always histologically homogeneous [12]. In addition, the present report shows that CCRCC tumors with more than 3.7 cm display ITH for Vimentin immunostaining. Interestingly, both size numbers are very close to the pathological staging frontier between pT1a and pT1b, suggesting that 4 cm in diameter is the size in which ITH appears visible on both histological and immunohistochemical settings. This finding, however, does not infer that ITH at gene mutation status level follows the same tendency.

The present work was also designed to investigate the utility of immunohistochemistry in the identification of ITH in CCRCC. The panel of protein markers used was selected considering their potential roles in the development of tumor aggressiveness, tumor diversification and metastatic processes.

EMT is a cellular mechanism extensively reviewed in the literature [3, 9, 20, 21]. The process explains how, under certain conditions, epithelial cells transform into mesenchymal cells. Briefly, EMT includes first the loss of the apical-basal polarity and cell-to-cell lateral junctions and then the acquisition of spindle cell shape, end-to-end polarity, and migration abilities. This change occurs in three different settings: embryonic development, tissue repair and neoplasia. The EMT concept is indispensable to understand the invasive properties and the metastatic capacities of carcinomas [22–27]. Interestingly, EMT may be a reversible process, what is called mesenchymal-to-epithelial transition (MET), which partially explains why high grade carcinomas may appear in distant metastases with an extremely low grade phenotype [22, 23, 28].

The immunohistochemical expression of several EMT markers in renal cell carcinomas has been previously reported. For instance, Clusterin and Twist expression has been related with tumor aggressiveness [29], tumor recurrence [30], and poor prognosis [29, 31]. Our study has focused on the immunohistochemical distribution of seven EMT markers [including cell-surface (E-cadherin) markers, cytoskeletal and cytoskeletal-associated (vimentin, β-catenin) proteins, and transcription factors (Snail, ZEB1, Twist, β-catenin)] in the context of ITH in CCRCC.

The Snail antibody tested in this study recognizes both Snail1 and Snail2. Snail1 is a zinc-finger transcription factor directly involved in the repression of E-cadherin transcription thus promoting the acquisition of a mesenchymal phenotype [28, 32]. Snail1 seems to be activated by ghrelin, an appetite-regulating molecule that promotes growth hormone release [33]. The immunohistochemical expression of Snail1 in CCRCC has been associated to advanced stage, high grade, local invasion and metastases [34]. A recent study has confirmed that Snail1 immunostaining predicts early recurrence and poor survival in CCRCC patients [35]. In our series, all high grade CCRCC tumors, including 3 G4 cases, displayed positive immunostaining for Snail. By contrast, Snail negative CCRCC were always low-grade. In addition, we have found that Snail positive immunostaining determines ITH, as defined by pathological analysis. The effect of Snail1 in promoting EMT can be suppressed by Klotho, as demonstrated by Zhu et al. in a recent study [36]. Klotho, an anti-aging and tumor suppressor protein expressed in renal tubular cells, inhibits PI3K/Akt/GSK3β/Snail signaling thus suppressing EMT, tumor invasion and migration. How the Snail1-associated transcriptional cell profile may be related with ITH in CCRCC remains to be investigated.

Twist is a transcription factor classically involved in embryonic development [37]. In addition, Twist plays a role in tumor growth, cell invasion and metastases by regulating neoangiogenesis and extracellular matrix degradation [29]. Twist expression is associated with bad prognosis in CCRCC [29, 30]. We have found heterogeneous Twist immunostaining in 16 CCRCC (40 %) and homogeneously positive staining in 1 case, although Twist was an irrelevant variable for classification of ITH and grade.

ZEB1 and ZEB2 are zinc-finger E-box binding transcription factors crucial for the activation of EMT [21]. *ZEB1* gene is up-regulated in primary renal tumors compared with their metastases, suggesting a reversal MET process in the metastatic seed [38]. The miRNA-200 family is directly involved in EMT and MET processes targeting the E-cadherin repressors ZEB1 and ZEB2 [28, 39]. Interestingly, CCRCC show down-regulation of all the members of this miRNA family [40], a fact that may be useful in the differential diagnosis of CCRCC [41, 42]. In our study, ZEB1 immunostaining was heterogeneous in 25 cases (62.5 %) and homogeneously positive in 1 case, but after attribute selection methods, ZEB1 immunostaining was a non-relevant variable for ITH classification as well as for tumor grade classification. However, a combined negative immunostaining for ZEB1 and Twist was always associated to low grade (G1/2) tumors, suggesting that the combined analysis of these two markers could be informative for CCRCC prognosis.

β-catenin is a cytoplasmic protein that links cadherins to cytoskeleton. Additionally, it serves as a co-transcriptional activator in the nucleus [43]. β-catenin is expressed in neoplastic cells undergoing EMT, especially at the stromal invasion front, as reported in colorectal adenocarcinoma [44]. β-catenin dysregulation has been associated to aggressive clinical course and shorter survival in CCRCC [45]. A heterogeneous immunohistochemical expression of this protein has been demonstrated in 15 cases in the present series, but this number has not reached any significance in the CART algorithms. In addition, β-catenin immunostaining was diffusely positive (nucleus and cytoplasmic membrane) in 22 cases and totally negative in 3 cases.

E-cadherin is expressed at the surface of epithelial cells in normal conditions, decreasing in abundance during EMT process in a switch with N-cadherin [46]. E-cadherin immunostaining is completely lost in 60 % of our cases. By contrast, focal and diffuse immunostaining was detected in 15 and 1 cases, respectively. After attribute selection methods, E-cadherin immunostaining was not a relevant variable for ITH.

Vimentin is an intermediate filament that has been implicated in all EMT scenarios (embryonal development, tissue repair and cancer progression) [28]. Low mRNA levels of Vimentin correlate with a better outcome of CCRCC patients [38]. From a practical viewpoint, Vimentin is usually co-expressed with cytokeratins and CD10, helping in the routine differential diagnosis of renal tumors [47]. The pathologist’s experience reveals that Vimentin immunostaining is sometimes patchy in CCRCC. In our study, Vimentin immunostaining was heterogeneous in half of the cases (20 cases), being homogeneously positive in 18 cases and totally negative in 2 cases. As previously mentioned, our classification analysis reveals a relation between tumor size larger than 3.7 cm and heterogeneous Vimentin immunostaining.

p110α, PTEN and p-Akt are major signaling components in the pathway leading to mTOR activation, and were included in our study because of their importance in the molecular profiling of CCRCC and their emerging utility as prognostic markers and therapeutic targets [48–51]. p110α is a major catalytic subunit of the PI3K enzyme and displays pro-oncogenic properties by virtue of generation of the phospho-inositide PIP3, which favors the phosphorylation and activation of the Akt effector kinase. Phospho-Akt (p-Akt) executes pro-oncogenic functions by phosphorylation of multiple protein substrates, being an important upstream activator of mTOR. This effect is counteracted by the action of the PTEN PIP3 phosphatase [52–54]. Most of the tumor samples analyzed in our study displayed p110α positive and PTEN negative immunostaining, in agreement with their respective oncogenic and tumor suppressive functions in renal cancer [55]. However, we did not find correlation between PTEN and p-Akt (as a measurement of Akt activation) immunostaining in most of the tumors, suggesting the existence of PTEN-independent mechanisms driving Akt phosphorylation and activation in CCRCC, as previously proposed by others [56]. Most cases (33/40) displayed homogenous positive staining for p110α, whereas the majority of samples were homogenously negative for PTEN (26/40) or p-Akt (28/40). Classification analysis with these markers was not relevant for ITH.

*SETD2* is the main methyltransferase responsible for trimethylation of histone-3 at lysine-36 and is encoded in chromosome 3, whose LOH has been reported to be an early event in CCRCC [4]. In spite of the fact that this gene is mutated in 4–8 % of CCRCC [3], there is no evidence that these mutations carry clinical significance [57]. Very recent studies have shown that *SETD2* loss of function causes dysfunctional DNA replication and repair, which promotes subclonal diversification. This contributes to ITH acting at early branches of the tumor phylogenetic tree [5]. We have detected a heterogeneous immunostaining of SETD2 protein in almost half of the cases (42.5 %), but SETD2 was an irrelevant variable for classification of IHT and grade.

## Conclusions

ITH is a common event in CCRCC, even in organ-confined tumors. Aside from the molecular approaches, conventional histologic and immunohistochemical studies may also demonstrate ITH in CCRCC, as revealed in this study. For such a purpose, Snail immunostaining appears to be an important marker. The high incidence of ITH in CCRCC recommends a wide sampling of the surgical specimens to guarantee that crucial data for the patient are not overlooked.

## Abbreviations

CART: Classification and regression tree algorithm
CCRCC: Clear cell renal cell carcinoma
EMT: Epithelial to mesenchymal transition
HE: Hematoxylin-eosin staining
ITH: Intratumor heterogeneity
LOH: Loss of heterozygosity
MET: Mesenchymal to epithelial transition
TMA: Tissue microarray
WEKA: Waikato Environment for Knowledge Analysis
